# Survey on surgical treatment of neonatal necrotizing enterocolitis in China 2022

**DOI:** 10.1136/wjps-2023-000588

**Published:** 2023-08-08

**Authors:** Jiafang Gao, Dengming Lai, Jinfa Tou

**Affiliations:** 1 Department of Neonatal Surgery, Children's Hospital, Zhejiang University School of Medicine, Hangzhou, China; 2 Neonatal Surgery Group of the Pediatric Surgery Branch, Chinese Medical Association, Beijing, China

**Keywords:** Child Health, Gastroenterology, Hospitals, Pediatric

## Abstract

**Objective:**

The aim of this study was to identify the state of surgical treatment of neonatal necrotizing enterocolitis (NEC) in China.

**Methods:**

A total of 246 delegates (88.0% senior surgeons) completed a survey sent by the Neonatal Surgery Group of the Pediatric Surgery Branch of the Chinese Medical Association in 2022. Five centers were eliminated due to lack of experience.

**Results:**

Generally, 38.2% of surgeons work in centers where more than 20 cases of surgical NEC are treated per year. A total of 81.3% of surgeons reported the use of ultrasonography; the most used biomarkers were white blood cell count (95.9%), C-reactive protein (93.8%), and procalcitonin (76.3%). Most surgeons (80.9%) used a combination of two (67.2%) antibiotics or single (29.5%) antibiotic for a treatment period of 7–14 days, and most used antibiotics were carbapenems (73.9%), penicillin and cephalosporins (56.0%). Patients are issued the fasting order for 5–7 days by nearly half surgeons (49.8%) for conservative treatment. 70.1% of surgeons deemed that the most difficult decision was to evaluate the optimal timing of surgery. Most surgeons (76.3%) performed diagnostic aspiration of peritoneal fluid. Laparoscopy was performed for the diagnosis and/or treatment of NEC by 40.2% of surgeons. A total of 53.5% of surgeons reported being able to identify localized intestinal necrosis preoperatively. Surgeons relied the most on pneumoperitoneum (94.2%) and failure of conservative treatment (88.8%) to evaluate the surgical indications. At laparotomy, surgical treatments vary according to NEC severity. Infants are fasted for 5–7 days by 55.2% of surgeons postoperatively. Most surgeons (91.7%) followed up with patients with NEC after discharge for up to 5 years (53.8%).

**Conclusions:**

The most difficult aspect of surgical NEC is evaluating the timing of surgery, and surgeons in the children’s specialized hospitals are experienced. The treatment of NEC totalis is controversial, and the indications for laparoscopy need to be further clarified. More multicenter prospective studies are needed to develop surgical guidelines in the future.

**WHAT IS ALREADY KNOWN ON THIS TOPIC**

- Controversy persists over the most appropriate way to manage necrotizing enterocolitis (NEC) worldwide.

**WHAT THIS STUDY ADDS**

- Current state of surgical treatment of NEC in China, especially evaluating the timing of surgery and use of laparoscopy.

**HOW THIS STUDY MIGHT AFFECT RESEARCH, PRACTICE OR POLICY**

- Calling for more multicenter prospective studies to develop surgical guidelines.

## Introduction

Necrotizing enterocolitis (NEC) is considered to be one of the most devastating inflammatory gastrointestinal diseases in newborns, with a high mortality rate of 20–30%, especially for surgical patients.^1 2^ With the improvement of neonatal-perinatal medicine, the incidence and mortality of NEC have decreased.^3^ However, epidemiological investigations have revealed the increased incidence of surgery for NEC, probably due to the higher rate of preterm and even ultra-premature births. It is difficult to reduce the mortality of surgical NEC, which is approximately 30% regardless of birth weight.^1 2 4^ Although studies have been conducted for decades, controversy persists over the most appropriate way to manage NEC.^4 5^

In 2015, the European Paediatric Surgeons Association published the ‘International Survey on the Management of Necrotizing Enterocolitis’ with the absence of data from China.^5^ As one of the countries with the largest number of newborns, it is necessary to conduct a study on the current state of NEC treatment to provide the latest opinions of pediatric surgeons in China.

## Methods

In October 2022, the Neonatal Surgery Group of the Pediatric Surgery Branch of the Chinese Medical Association conducted an anonymous electronic questionnaire survey among pediatric surgeons across the country. Respondents were asked to fill in their hospital, hospital degree, type of hospital, rank, number of infants with new surgical NEC enrolled each year, and department involved in NEC management (see the concrete content of the questionnaire in online supplemental materials).

10.1136/wjps-2023-000588.supp1 Supplementary data

The questionnaire focused on the results of preoperative imaging and laboratory examination, conservative treatment, timing of surgery, surgical treatment, and short-term and long-term postoperative management. In terms of the timing of surgery, we define failure of conservative management as when non-surgical treatment is ineffective for abdominal distension and bloody stools worsen, indicators of peritonitis appeared and worsen, an abdominal X-ray revealed fixed intestinal loops, or laboratory tests revealed severe infections, acidosis, and electrolyte imbalances.

According to the Clinician Self-Administered Survey Design and Implementation Guidelines, we performed predictive and clinical sensibility tests before issuing the questionnaire.^6^

This questionnaire was completed by surgeons from 246 different centers, and we selected the most representative questionnaire for each center based on the rank. Five centers were eliminated due to lack of experience. The final statistical analysis included 241 participants, including 114 chief physicians, 98 deputy chief physicians, and 29 attending physicians. There were 135 comprehensive hospitals and 106 specialized children’s hospitals in 241 centers, including 203 grade A tertiary hospitals.

In the result of ‘surgical options according to NEC severity’, we compared the difference between China and Europe based on the data obtained from the ‘International Survey on the Management of Necrotizing Enterocolitis’.^5^

## Results

### Centers

This questionnaire covered a total of 22 provinces, 4 municipalities and 5 autonomous regions, including almost the entire Chinese mainland. In general, 82 (34.0%) respondents worked in a center that treats fewer than 10 NEC cases a year, 67 (27.8%) worked in a center that treats 10–20 NEC cases, and 52 (21.6%) worked in a center that treats 20–50 NEC cases. Only 40 (16.6%) respondents worked in a center that treats more than 50 NEC cases a year.

Most centers (139 respondents, 57.7%) reported that NEC was managed by departments of neonatal surgery and neonatology, including the neonatal intensive care unit (NICU). With regard to those who were managed by a single department, 67 respondents (27.8%) referred to the department of neonatology (including the NICU), whereas smaller proportions of them indicated the department of neonatal surgery (23, 9.5%) and department of general surgery alone (12, 5.0%).

### Assistant examination

If NEC was suspected, 229 (95.0%) surgeons ordered a routine abdominal X-ray, and 196 (81.3%) surgeons ordered an intestinal Doppler ultrasonography. The most commonly featured abdominal signs in X-ray and Doppler ultrasonography were reported (online supplemental figures 1 and 2). Surgeons mainly focused on pneumatosis intestinalis (217, 94.8%), pneumoperitoneum (216, 94.3%), and portal venous gas (194, 84.7%) in X-rays, while the primary concerns of Doppler ultrasonography were seroperitoneum (183, 93.4%), pneumatosis intestinalis (174, 88.8%), and portal venous gas (158, 80.6%), which can be used to diagnose and monitor the severity of NEC.

Moreover, 63 (26.1%) surgeons reported the use of abdominal CT, whereas only nine surgeons reported the use of abdominal MRI scans.

**Figure 1:**
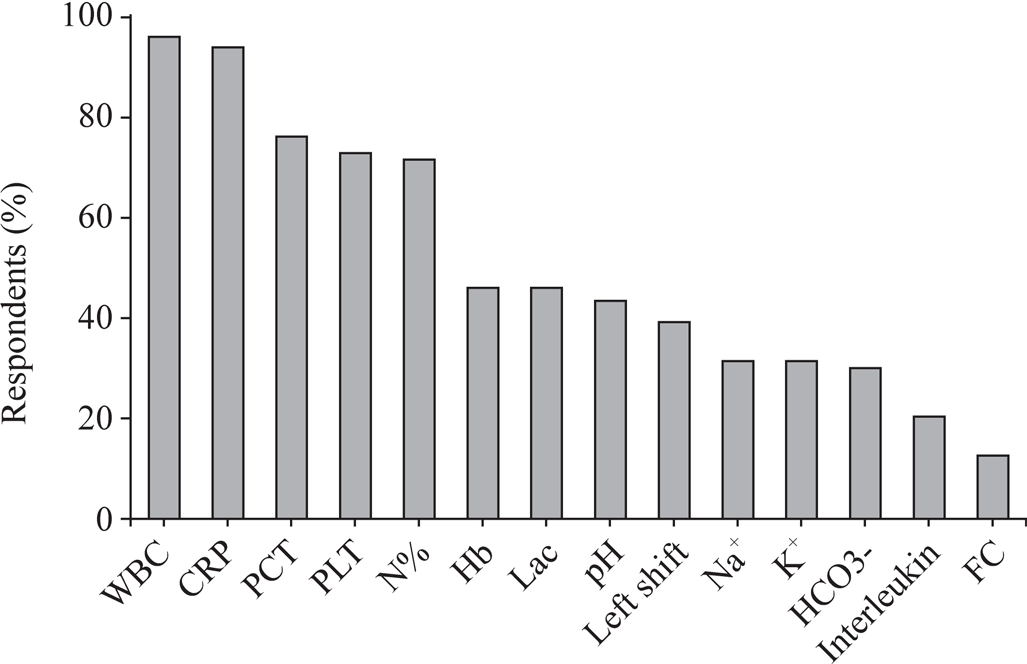
The most commonly used biochemical markers for diagnosis and monitoring of NEC. CRP, C-reactive protein; FC, fecal calprotectin; Hb, hemoglobin; Lac, lactate; NEC, necrotizing enterocolitis; N%, neutrophil percentage; PCT, procalcitonin; PLT, platelets; WBC, white blood cell count.

In the diagnosis and monitoring of NEC, surgeons mainly relied on white blood cell count (WBC; 95.9%), C-reactive protein (CRP) concentration (93.8%), procalcitonin (PCT; 76.3%), platelet count (72.6%), and neutrophil percentage (71.8%) (figure 1). Less commonly used biochemical markers included fecal calprotectin (12.4%), interleukin (IL; 20.7%), and HCO_3_− (30.3%).

### Non-operative treatment

For medically managed NEC, 117 (48.5%) surgeons administered antibiotics for 7–10 days, and 78 (32.4%) preferred to administer antibiotics for 11–14 days. A small number of respondents provided their antibiotic therapy for longer or shorter periods of time, including 21 (8.7%) for up to 7 days and 25 (10.4%) for more than 2 weeks (online supplemental figure 3). The amounts of antibiotics were unevenly distributed as follows: single by 71 (29.5%) surgeons, combination of two by 162 (67.2%), and three by 7 (2.9%). One respondent even used the combination of four antibiotics. During treatment, surgeons prioritized carbapenems (73.9%), penicillin and cephalosporins (56.0%), followed by nitroimidazoles (39.4%) and glycopeptides (20.3%). Aminoglycosides and antifungal drugs are rarely used (online supplemental figure 4).

Patients are fasted for less than 5 days by 14 (5.8%) responders, 5–7 days by 120 (49.8%), 8–10 days by 66 (27.4%), and more than 10 days by 41 (17.0%) respondents (online supplemental figure 5).

### Timing of surgery

Of all the responding surgeons, 184 (76.3%) performed diagnostic abdominal paracentesis (DAP) in patients with suspected intestinal perforation with ascites. Two-fifths of surgeons (97, 40.2%) would consider laparoscopy for the diagnosis and/or treatment of NEC.

In evaluating surgical indications for NEC, surgeons relied most on pneumoperitoneum (227, 94.2%) and failure of conservative treatment (214, 88.8%), and laid less emphasis on pneumatosis intestinalis and portal venous gas (143, 59.3%) and seven clinical metrics of metabolic derangement (MD7) ≥3 (116, 48.1%).^7 8^ Furthermore, 46 (19.1%) respondents reported greater than 90% certainty in identifying the presence of localized intestinal necrosis preoperatively (before intestinal perforation), while 83 (34.4%), 84 (34.9%), and 28 (11.6%) reported another three levels of certainty: three-quarters, half and less than one-quarter.

The majority (169, 70.1%) of surgeons reported that evaluating the optimal timing of surgery was the most difficult point in the treatment of NEC, followed by successful postoperative intensive treatment and complication prevention.

### Surgical operation

**Figure 2:**
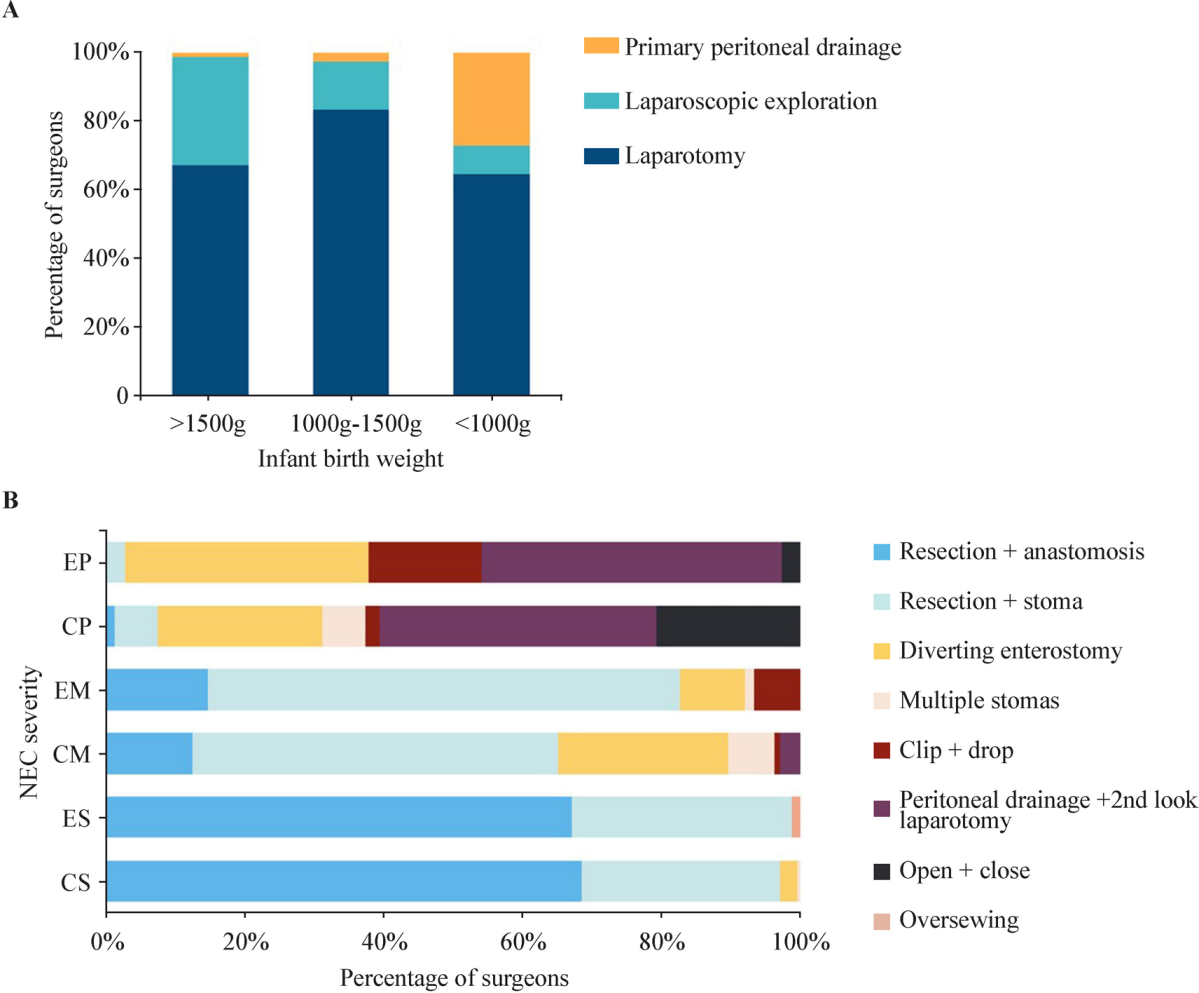
(A) Surgical options according to necrotizing enterocolitis (NEC) infants’ weight. (B) Difference in surgical options according to NEC severity between China and Europe. CM, data of multiple perforations or areas of necrosis in China; CP, data of panintestinal NEC in China; CS, data of a single perforation or area of necrosis in China; EM, data of multiple perforations or areas of necrosis in Europe; EP, data of panintestinal NEC in Europe; ES, data of single perforation or area of necrosis in Europe.

In infants weighing more than 1500 g, 162 (67.2%) surgeons performed a laparotomy, 76 (31.5%) performed laparoscopic exploration, and 3 (1.2%) preferred primary peritoneal drainage (PPD). In very low birthweight infants, 201 (83.4%) surgeons performed a laparotomy, 34 (14.1%) performed laparoscopic exploration, and 6 (2.5%) preferred PPD. In extremely low birth weight (ELBW) infants, most (156, 64.7%) surgeons preferred a laparotomy, laparoscopic exploration and PPD were performed by 20 (8.3%) and 65 (27.0%) surgeons, respectively (figure 2A).

Surgical options vary on the basis of NEC severity at laparotomy. In the case of single perforation or area of necrosis, 165 (68.5%) surgeons performed bowel resection and anastomosis, 69 (28.6%) performed enterostomy, few respondents (6, 2.5%) created a diverting enterostomy, and only one surgeon created multiple stomas. Where there are multiple perforations or areas of necrosis, 127 (52.7%) surgeons created a stoma, 59 (24.5%) performed a diverting enterostomy, 30 (12.4%) performed bowel resection and anastomosis, 16 (6.6%) created multiple stomas, few (7, 2.9%) performed peritoneal drainage and a second-look laparotomy, and only two performed clip and drop technique. In the case of panintestinal NEC, 96 (39.8%) surgeons performed peritoneal drainage and a second-look laparotomy, 57 (23.7%) performed diverting enterostomy, 50 (20.7%) closed the abdomen without implementing any procedure, 15 (6.2%) created one or multiple stomas, 5 (2.1%) performed clip and drop technique, and 3 (1.2%) performed bowel resection and anastomosis (figure 2B).

Regarding stoma creation, 134 (55.6%) surgeons preferred the double barrel, 58 (24.1%) preferred the single barrel, 42 (17.4%) preferred the T-type stoma (Santulli stoma or Bishop-koop stoma), and 7 (2.9%) preferred the separated stoma. Moreover, 113 (46.9%) surgeons placed the stoma within the wound, and 128 (53.1%) made another stoma incision.

After enterostomy, 6 (2.5%) surgeons performed stoma closure on patients within 2 months, 149 (61.8%) between 2 and 4 months, 67 (27.8%) between 4 and 6 months, and 19 (7.9%) over half a year. A total of 227 (94.2%) surgeons performed routine bowel radiography before stoma closure; 99.1% of them evaluated distal intestinal stenosis, 92.1% assessed peristalsis of the distal intestine, and 77.1% estimated the length of the intestine.

### Postoperative management

After the surgery, infants were fasted for 5–7 days by 133 (55.2%) surgeons, 8–10 days by 50 (20.7%), less than 5 days by 39 (16.2%) and more than 10 days by 19 (7.9%). A total of 103 (42.7%) surgeons restarted infants on hydrolyzed formulas, 72 (29.9%) on breast milk, 36 (14.9%) on amino acid-based formulas, and 15 (6.2%) on premature formula and standard formula separately.

Most (221 respondents, 91.7%) centers followed up surgical patients after hospital discharge. More than half (144, 65.2%) surgeons reported that patients were followed up in the clinics of pediatrics and pediatric surgery, 59 (26.7%) reported follow-up in the clinic of pediatric surgery alone, and 18 (8.1%) in the clinic of pediatrics alone. The follow-up duration lasted no more than 1 year in 89 (40.3%) centers, up to 5 years in 119 (53.8%) centers, and beyond 5 years in 13 (5.9%) centers.

## Discussion

This survey of practice reflected the general level of NEC surgical treatment provided in China at present and brought to light the lack of consensus on certain NEC management issues. As many of the current NEC definitions include subjectively chosen criteria, multicenter prospective studies, ideally on a global scale, should be conducted to develop a consensus definition.

**Figure 3:**
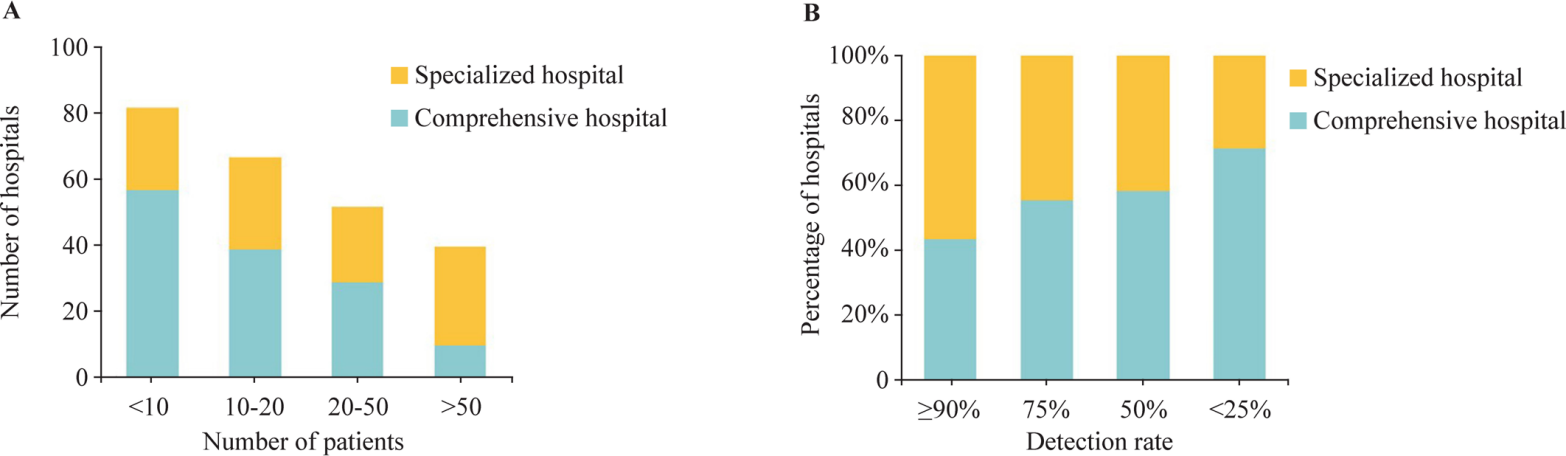
(A) The number of children’s specialized and comprehensive hospitals with different numbers of patients with necrotizing enterocolitis (NEC) enrolled per year. (B) The relative percentage of children’s specialized and comprehensive hospitals with different certainty of identifying the presence of localized intestinal necrosis preoperatively (before intestinal perforation).

In China, patients with NEC are more concentrated in large central specialized hospitals, as seen by the higher annual admission volumes of patients with NEC for these hospitals in our survey (figure 3A). NEC is co-managed by the department of neonatal surgery and department of neonatology in most centers, and multidisciplinary treatment is currently adopted since it offers greater benefits.

Growing evidence has demonstrated that abdominal radiographs and abdominal ultrasound (AUS) are both the cornerstones of NEC diagnosis and management; nevertheless, the first-line use of AUS has been minimal.^9^ Recently, surgeons have gradually realized the importance of AUS in NEC, and AUS is more sensitive and can predict whether surgery is needed earlier to improve the prognosis.^10–12^ A total of 81.3% of surgeons reported the use of AUS in this survey, which was higher than that reported previously.^5 9 10^ The signs that surgeons concern are typically those relate to the timing of operation, such as portal venous gas. In the future, more standardized ultrasound examination or a more accurate diagnostic model combined with ultrasound may be beneficial.^9–11^ Although a CT scan is not a suitable choice for neonates, 26.1% of surgeons ordered it.

Neutropenia, thrombocytopenia, metabolic acidosis, and elevated CRP levels are common signs in infants with NEC.^7 8^ The great majority of surgeons indeed relied on these parameters for the assessment of severity in our study. It is noteworthy that surgeons have paid less attention to hyponatremia because it is an independent predictor of bowel ischemia and reflects the severity of inflammation.^7 13^ Interpretation of electrolyte disorders should be given more attention.^13^ WBC, CRP, PCT, and IL-6 are all non-specific mediators of inflammation; CRP is the most commonly used biomarker as a late-warning biomarker, whereas IL-6, an early-warning biomarker, has not been frequently employed in clinical settings because many hospitals do not have relevant detection capability.^14 15^ Since the cut-off values for fecal calprotectin cannot be identified and it is significantly influenced by gestational and postnatal age, it is rarely used in routine clinical practice.^14^

No agreement has been achieved about the empirical use of broad-spectrum antibiotics for medical NEC, which is also represented in the questionnaire. According to the severity of the disease, antibiotics should be applied for 7–14 days, varying from 7-10 days for mild cases to 10–14 days for severe cases.^16 17^ Currently, ampicillin, gentamycin, and metronidazole are widely used abroad, while carbapenems, penicillin and cephalosporins, and nitroimidazole are the most used antibiotics in China.^17 18^ There is no consensus on the best combination of antibiotics.^19^ Aminoglycosides are not commonly used in China due to ototoxicity and nephrotoxicity. Another essential component of treatment is fasting, which is typically advised for 7–10 days.^1^ Some surgeons would choose to fast for a shorter period of time. Currently, some studies indicate that fasting for fewer than 7 days does not increase the risk of NEC recurrence, intestinal stenosis, or mortality in infants with non-surgical NEC.^1 20 21^

A proportion of NEC infants ultimately require surgical intervention. In this survey, it is considered that the judgment of the timing of the surgery is the most challenging. For patients with NEC who do not have intestinal perforation or who have a small anount of pneumoperitoneum but have an uncertain intestinal perforation, DAP is recommended to make a certain diagnosis to help determine the timing of surgery.^22^ Several results are commonly specific to surgical exploration: (1) fecal ascites which indicates intestinal perforation, (2) hemorrhagic one which even implies intestinal necrosis, and (3) purulent one which reminds severe abdominal infection and peritonitis.^22^ Pierro *et al* originally proposed the diagnostic use of laparoscopy for infants with NEC, and he suggested that laparoscopy provides information regarding intestinal viability, which can guide further surgical management even in critically ill neonates weighing less than 1000 g.^23^ Some case reports and animal models support this opinion.^24 25^ Surprisingly, pediatric surgeons are enthusiastic about laparoscopy in China, with 40.2% of surgeons considering laparoscopy for the diagnosis and/or treatment of NEC, which is significantly higher than the international level.^5^ The indications for laparoscopy in NEC are still unclear, and evidence-based guidelines are currently absent. At present, it is only clear that laparoscopy should not be performed on newborns who have unstable circumstances. It is also questionable whether the surgeons performed laparoscopic surgery too early, which was not the appropriate timing of this intervention for NEC.

Pneumoperitoneum and failure of conservative treatment are absolute surgical indications, and pneumatosis intestinalis and/or portal venous gas and MD7 ≥3 are relative surgical indications.^7 26^ Despite the fact that pneumatosis intestinalis and portal venous gas are the two most significant signals that surgeons rely on in imaging examination, they were not used widely to estimate the timing of surgery. This might be because imaging examinations, particularly AUS, are subjective and need to be performed at certain times, which results in frequent re-examination. Once intestinal necrosis has been diagnosed, surgery should be performed as quickly as feasible to treat the acute phase of NEC.^26^ The lack of specialized equipment forces surgeons to rely on their clinical experience to evaluate whether the intestine is necrotic. Surgeons in large-scale, specialized hospitals are more confident in their ability to detect localized intestinal necrosis preoperatively (before intestinal perforation) (figure 3B).

Regardless of the infant’s weight, most surgeons opt to perform an exploratory laparotomy. Furthermore, larger proportions of surgeons choose laparoscopic exploration for higher weights and PPD for lower weights. PPD is typically used on ELBW infants who cannot withstand laparotomy exploration; however, it is still controversial whether PPD would improve the prognosis compared with laparotomy exploration.^27 28^ According to our survey, 26.97% of surgeons use PPD in their practice for ELBW infants.

Despite personal experience, there have been several instructive principles of surgery in NEC: removal of necrotic intestine, control of intra-abdominal infection, and preservation of residual intestinal length to the greatest extent.^29^ Nevertheless, surgical strategy varies on the basis of surgeons’ preference and perspectives. The majority of surgeons choose bowel resection and anastomosis in cases of a single perforation or area of necrosis, regardless of China or Europe, whereas stoma formation predominates in cases with multiple perforations or areas of necrosis. Currently, bowel resection followed by stoma is the most recognized and feasible surgical method.^26^ NEC totalis remains the most challenging and controversial. Additionally, surgeons in China rarely perform the clip and drop technique, which is frequently used in Europe.

The type and location of the stoma are also up for debate.^30 31^ The double barrel reduces the risk of subsequent prolapse and may represent the preferred approach.^30^ Although the timing of stoma closure should be individualized depending on the infant’s weight, we usually recommend 6–12 weeks since stoma formation.^32^ Early stoma reversal at lower weight may be acceptable because of complications such as failure to thrive. Most surgeons opt for a contrast examination of the distal bowel prior to surgery, which assists them in estimating the bowel’s morphology and continuity.

In the postoperative period, early feeding promotes intestinal adaptation. A meta-analysis concluded that early feeding within 7 days is safe, and the risk of NEC recurrence and intestinal strictures will not increase.^21 33–35^ Following surgery, human milk is recommended as the first choice for nutrition in newborns.^35 36^ Extensively hydrolyzed formula is particularly advised for infants with who are intolerant of human milk.^37^ In the absence of human milk, the best type of formula milk to use is still debatable, and some experts recommend hydrolyzed formulas.^37–39^ Bovine milk-based products may increase the risk of NEC recurrence.^38^ In general, no specific implementation protocol is available.

Following discharge, NEC survivors have the risk of developing various medical and surgical conditions, including intestinal stenosis and failure to thrive. It is recommended that the patients should be monitored jointly by the departments of neonatal surgery and neonatology. Most surgeons uppatients, but not for a long period, so neurodevelopmental outcomes do not receive the intended attention.^40^

There are some limitations in our study. Although our survey has guiding significance for the treatment of surgical NEC in China, it cannot be used as evidence-based medical evidence. We explored the diagnostic and surgical options for NEC. Laparoscopic exploration is a prevalent diagnostic and treatment approach in China; however, its application, indications, and benefits still need to be researched more through multicenter prospective studies in the future.

In conclusion, the most challenging aspect of surgical NEC is evaluating the timing of surgery. Relative surgical indications for NEC have not yet been agreed, but specialized hospitals are relatively more experienced. It is worth noting that AUS has been extensively used in China, which will help to better judge the timing of surgery in the future. In surgical aspects, the surgical treatment for NEC totalis is controversial, and the indications for laparoscopic surgery need to be further clarified. In China, few patients with NEC are followed up for more than 5 years after discharge, and neurodevelopmental outcomes do not receive enough attention. Surgical NEC and medical NEC differ little in feeding, antibiotic therapy and so on. More multicenter prospective studies are needed in the future to develop surgical guidelines for NEC.

## Data Availability

Data are available upon reasonable request.
