# Supplementary material for: Survey on surgical treatment of neonatal necrotizing enterocolitis in China 2022
Source: World J Pediatr Surg. 2023 Aug 8;6(4):e000588. doi: 10.1136/wjps-2023-000588 (PMC10414103; doi:10.1136/wjps-2023-000588)
Supplement: Supplementary data [file wjps-2023-000588supp001.pdf]

Supplemental Material

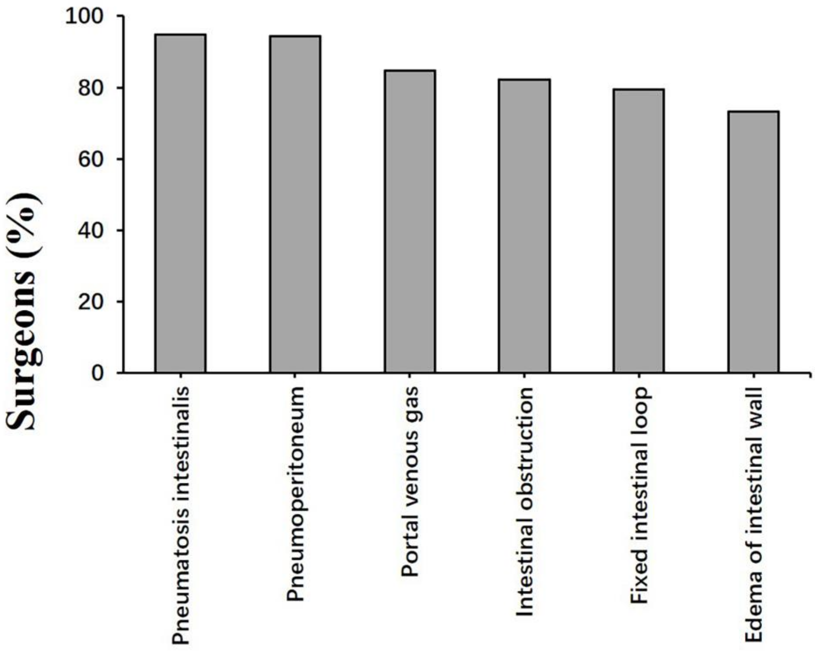

S. Fig. 1 The most commonly used abdominal signs in X-ray

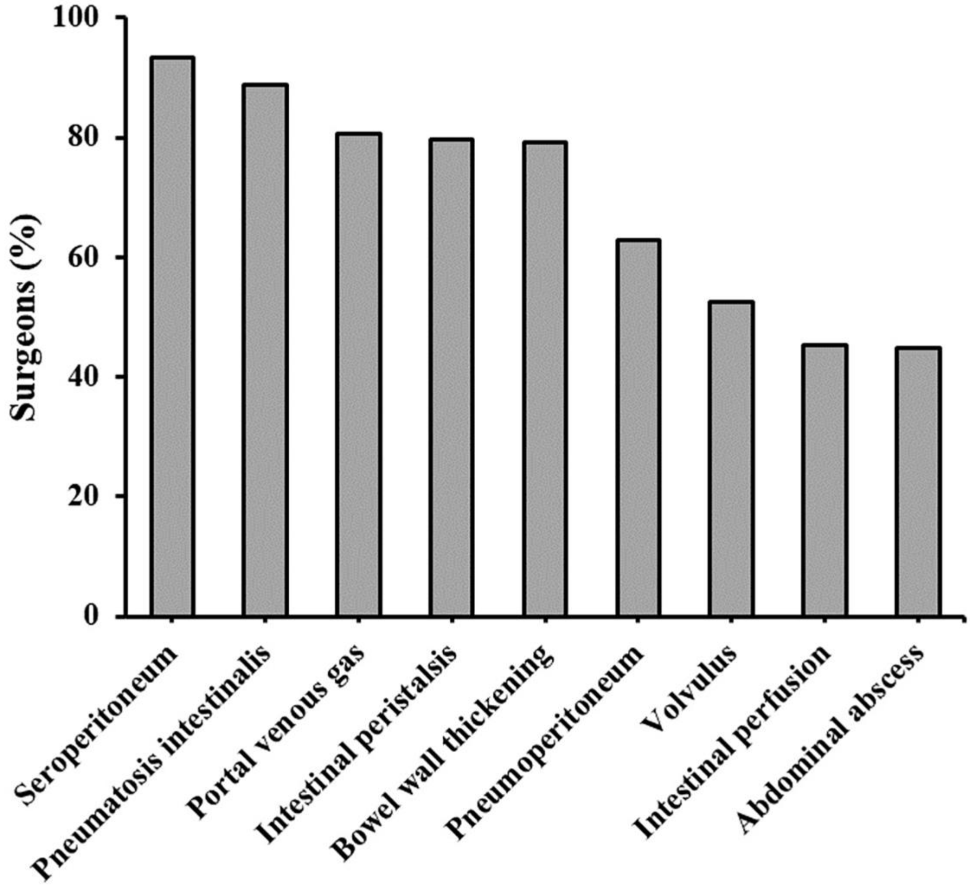

S. Fig. 2 The most commonly used abdominal signs in Doppler ultrasonography.

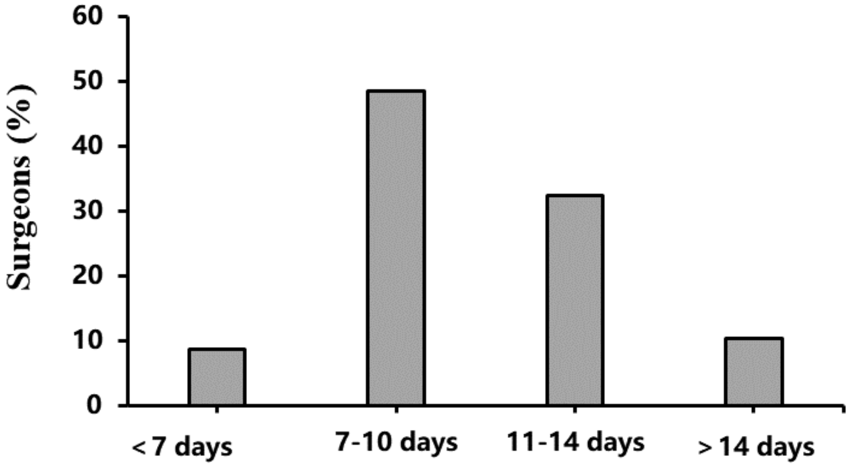

S. Fig. 3 The period of antibiotics administration in conservative treatment

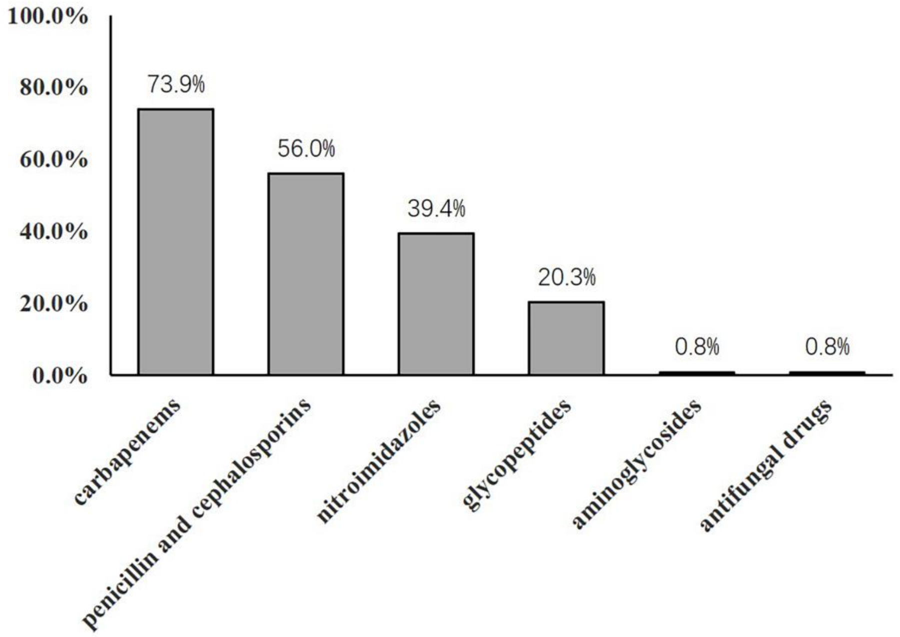

S. Fig. 4 The category of antibiotics uesd in conservative treatment

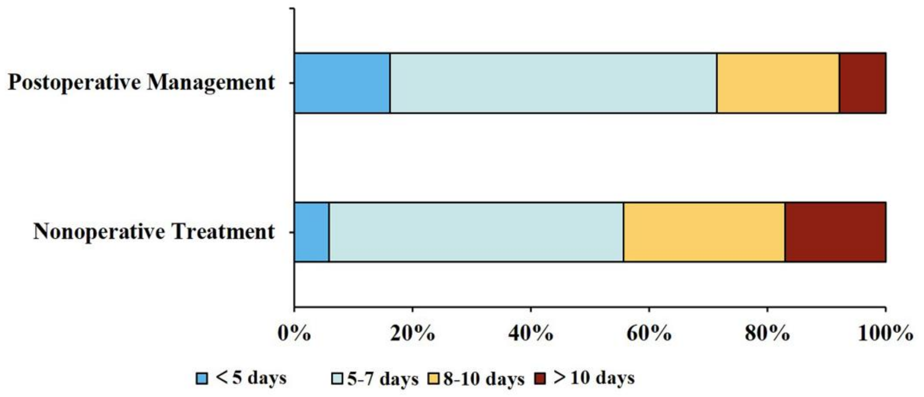

S. Fig. 5 Fasting time for nonoperative treatment and postoperative management

Questionnaire

Basic information

1. What is the full name of your hospital?
2. Which type of hospital do you work in?
   A. comprehensive hospital
   B. children's specialized hospital
3. What is the level of your hospital?
   A. Grade-A tertiary hospital
   B. Grade-B tertiary hospital
   C. Grade-A secondary hospital
   D. Grade-B secondary hospital
4. What is your professional title?
   A. chief physician
   B. deputy chief physician
   C. attending
   D. resident
5. How many new cases of infants with surgical NEC enrolled each year in your hospital?
   A. ＜10
   B. 10-20
   C. 20-50
   D. ＞50
6. Which departments involved in NEC management in your hospital?
   A. Departments of neonatal surgery
   B. Department of neonatology (including NICU)
   C. Department of general surgery
   D. A+B

Assistant Examination

1. (MCQ) When NEC is suspected, what kind of imaging examinations would you order?
   A. Abdominal X-ray
   B. Intestinal doppler ultrasonography
   C. MRI
   D. CT
2. (MCQ) Which signs will you focus on more commonly in abdominal X-ray?
   A. Pneumatosis intestinalis
   B. Pneumoperitoneum
   C. Portal venous gas
   D. Intestinal obstruction
   E. Fixed intestinal loop
   F. Edema of intestinal wall

3. (MCQ) Which signs will you focus on more commonly in doppler ultrasonography?
   A. Seroperitoneum
   B. Pneumatosis intestinalis
   C. Portal venous gas
   D. Intestinal peristalsis
   E. Bowel wall thickening
   F. Pneumoperitoneum
   G. Volvulus
   H. Intestinal perfusion
   I. Abdominal abscess
4. (MCQ) During the diagnosis and monitoring of NEC, which biomarkers do you rely on most commonly?
   A. WBC
   B. CRP
   C. PCT
   D. PLT
   E. N%
   F. Hb
   G. Lac
   H. pH
   I. Left Shift
   J. Na^+
   K. K^+
   L. HCO_3^-
   M. Interleukin (IL-6, etc)
   N. FC

Nonoperative Treatment

1. How long do you usually administrate antibiotics?
   A. ＜7 days
   B. 7-10 days
   C. 11-14 days
   D. ＞14 days
2. How many kinds of antibiotics do you usually administrate?
   A. 1
   B. 2
   C. 3
   D. 4
3. (MCQ) What many kinds of antibiotics do you frequently use?
   A. Carbapenems
   B. Penicillin and cephalosporins
   C. Nitroimidazoles

D. Glycopeptides
E. Aminoglycosides
F. Antifungal drugs

4. How long does the fasting take under conservative treatment for NEC?

A. ＜5 days
B. 5-7 days
C. 8-10 days
D. ＞10 days

Timing of Surgery

1. Will you choose to perform diagnostic abdominal paracentesis (DAP) for patients with suspected intestinal perforation and ascites?
   A. Yes
   B. No
2. Will you consider laparoscopy for diagnosis and/or treatment of NEC?
   A. Yes
   B. No
3. (MCQ) What is the surgical indication that you usually depend on in the assessment in NEC?
   A. Pneumoperitoneum
   B. Failure of conservative treatment
   C. Pneumatosis intestinalis and portal venous gas
   D. MD7 (seven clinical metrics of metabolic derangement) ≥3
4. To which extent of certainty will you identify the presence of localized intestinal necrosis preoperatively (before intestinal perforation)?

A． ≥90%

B．75%

C．50%

D． ＜25%

Surgical Operation

1. Surgical options according to NEC infants' weight:

A． Laparotomy

B．Laparoscopy

C．Primary peritoneal drainage (PPD)

Which surgical option will you choose for infants with NEC weighting more than 1,500g?

Which surgical option will you choose for infants with NEC weighting between 1,000–1,500g?

Which surgical option will you choose for infants with NEC weighting less than 1,000g?

2. Surgical options according to NEC severity at laparotomy:
   A. Resection + anastomosis
   B. Resection + stoma
   C. Diverting enterostomy
   D. Multiple stomas
   E. Clip + drop
   F. Peritoneal drainage + 2nd look laparotomy
   G. Open + close

   Which surgical option will you choose in the situation of intestinal perforation or necrosis involved a single area?

   Which surgical option will you choose in the situation of intestinal perforation or necrosis involved several areas?

   Which surgical option will you choose in the matter of pan intestinal NEC?
3. Which kind of stoma do you commonly adopt?
   A. Double barrel
   B. Single barrel
   C. T -type stoma (Santulli stoma or Bishop -hoop stoma)
   D. Separated stoma
4. Which site do you prefer for the stoma?
   A. Place the stoma within the existing incision
   B. Make another incision for stoma
5. Do you perform gastrointestinal radiography routinely before stoma closure?
   A. Yes
   B. No
6. (MCQ) What are your main concerns for evaluation before stoma closure?
   A. Distal intestinal stenosis
   B. The peristalsis of distal intestinal
   C. The length of intestine
7. How soon do you typically perform stoma closure after enterostomy?
   A. ＜2months
   B. 2-4 months
   C. 4-6 months
   D. ＞6months

Postoperative Management

1. How long does the fasting usually take postoperatively?
   A. ＜5 days
   B. 5-7 days

C. 8-10 days

D. ＞10 days

2. Which type of formula is your first choice when restarting feeding postoperatively?
   A. Breast milk
   B. Standard formula
   C. Hydrolyzed formula
   D. Amino acid-based formula
   E. Premature formula
3. Does your hospital provide follow-up for NEC patients?
   A. Yes
   B. No
4. If you choose “Yes”, how long does the follow-up period take?

   A. ＜1year

   B. 1-5 years

   C. ＞5years

5. Which department is mainly responsible for the follow-up?
   A. Pediatrics
   B. Pediatric Surgery
   C. A+B
6. What is the most difficult point in the management of NEC?
   A. Diagnose of NEC
   B. Evaluating optimal timing of surgery
   C. Infection control
   D. Postoperative nutrition management
   E. Postoperative intensive treatment
   F. Precaution of complications.
